# Intra-articular injection PLGA blends sustained-release microspheres loaded with meloxicam: preparation, optimization, evaluation *in vitro* and *in vivo*

**DOI:** 10.1080/10717544.2022.2144545

**Published:** 2022-11-11

**Authors:** Zheng Sun, Xuejing Gu, Teng Hao, Jiali Liu, Rongrong Gao, Yanli Li, Bin Yu, Hui Xu

**Affiliations:** School of Pharmacy, Collaborative Innovation Center of Advanced Drug Delivery System and Biotech Drugs in Universities of Shandong, Key Laboratory of Molecular Pharmacology and Drug Evaluation (Yantai University), Ministry of Education, Yantai University, Yantai, China

**Keywords:** Meloxicam, microsphere, PLGA blends, topical administration, osteoarthritis, anti-inflammatory

## Abstract

Meloxicam (MLX) is a commonly used drug in the clinical treatment of osteoarthritis, but it is associated with gastrointestinal adverse reactions. Therefore, in this study, we developed a sustained-release microsphere formulation of MLX for topical administration of knee joint. The MLX-loaded PLGA microspheres (MLX-MS) were prepared by emulsion solvent evaporation method with optimization of formulation using orthogonal experimental design. Physicochemical characterization results show MLX-MS were spherical with a smooth surface, the particle size was about 100 μm, drug loading was 30%, and encapsulation efficiency was 76.8%. In addition, the *in vivo* pharmacokinetics, tissue distribution, and pharmacodynamics were evaluated in rats by intra-articular administration of MLX. The microspheres showed a typical long-term sustained release pattern with a low initial burst release. In contrast to oral administration, local injection of MLX-MS produced a much higher value of elimination half-life time(T_1/2_) and peak time (T_max_) in plasma, while the intestinal drug distribution was significantly decreased. MLX-MS could also cause a greater reduction in the body level of IL-6 and TNF-α, which was positively correlated with R^2^=0.981. A good linear relationship (R^2^ = 0.9945) between the *in vitro* and *in vivo* drug release from MLX-MS could be observed, bivariate correlation analysis. All the findings demonstrated that local administration of MLX-MS can prolong the action time of MLX and reduce side effects, thus would be a promising preparation for the treatment of arthritis.

## Graphical Abstract

**Figure:**
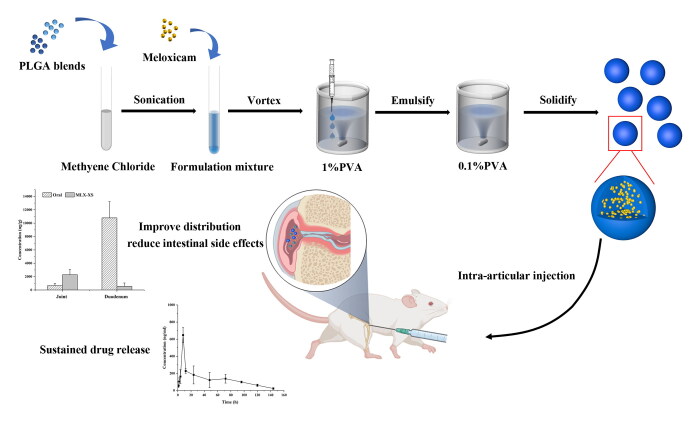

## 1. Introduction

Osteoarthritis (OA) is a common complex and multi-etiologic degenerative joint disease, which is characterized by a series of degenerative diseases such as joint local inflammation, cartilage degradation, and subchondral bone sclerosis. The global incidence rate is more than 3%, which is a serious threat to human health (Cross et al., 2014; Hanafy, & El-Ganainy, 2020). At present, there is still a lack of radical treatment for OA. In addition to surgical intervention, non-steroidal anti-inflammatory drugs (NSAIDs) are often used in clinical practice to alleviate arthritis-related swelling, stiffness, and pain. However, gastrointestinal reactions and other side effects are widespread and may increase the risk of cardiovascular disease (Laavola et al., 2018; Tang, 2019). Among them, meloxicam (MLX) is a selective inhibitor of cyclooxygenase (Cox)-2. Its intensity of action on Cox-2 in vitro is more than 10 times of Cox-1, which is lower than the risk of gastrointestinal mucosal injury caused by other NSAIDs and is commonly used in clinical symptoms of OA. Nevertheless, the conventional preparations of MLX still have serious adverse reactions such as gastric bleeding, headache, and rash when they are administered orally with high doses and long-term treatment (Patoia et al., 1996).

Compared with oral administration, local administration can not only reduce the side effects on the gastrointestinal tract but also avoid the first-pass effect and improve the bioavailability of the system. Given the use of NSAIDs in the treatment of mild to moderate OA pain, the European Union against Rheumatism (EULAR) and the National Institute for Health and Nursing Excellence (NICE) recommended the treatment plan of local administration before oral administration (Rodriguez-Merchan, 2018). Therefore, NSAID long-acting sustained-release drug delivery system for local administration has attracted extensive research attention.

Microspheres, as a new drug delivery system, usually refer to a particle dispersion system formed by drug adsorption or dispersion in the polymer matrix, with a diameter range of 1–1000 μm (Karan et al., 2020). Because of its rich structure and function, it has many advantages. For example, it can be administered in various ways, including subcutaneous injection, oral administration, and lung administration as an inhalation (Su et al., 2021; Chen & Fang, 2000). Moreover, as a commonly used drug delivery system, it can prolong the action time of drugs in the body, reduce the number of administrations, and improve the compliance of patients (Singh Malik et al., 2016). Poly (lactic-co-glycolic acid) (PLGA) is one of the most widely used drug delivery carriers, which has good biocompatibility and biodegradability. Therefore, the preparation of meloxicam sustained-release microspheres with PLGA carrier for local administration in the treatment of arthritis has good safety, which not only can achieve the long-term anti-inflammatory effect but also can reduce the gastrointestinal adverse reactions of NASID.

In this study, the S/O/W emulsion-solvent evaporation method was used to prepare MLX long-term sustained-release microspheres (MLX-MS) suitable for local injection with PLGA as the carrier. The key prescription factors and preparation process were screened and optimized, and the obtained microspheres were systematically characterized in vitro and evaluated in drug release characteristics, providing an experimental basis for the development of new preparations suitable for OA clinical practice.

## 2. Material and methods

### 2.1. Reagent and animals

MLX was purchased in Shandong Chenghui Shuangda Pharmaceutical Co., Ltd.; PLGA was provided by Shandong Lvye Pharmaceutical Co., Ltd. Polyvinyl alcohol (PVA) was purchased from German EMD Company; other reagents are from the national pharmaceutical chemical reagent company.

Male Wistar rats (200 ± 10 g, SCK20190003) were supplied by Jinan Pengyue Laboratory Animal Breeding Co., Ltd. All animals are strictly handled following the Care and Use of Laboratory Animals (NIH Publication No. 85-23, revised 1996) and approved by the Animal Experimentation Ethics Committee of Yantai University (No. 20220302).

### 2.2. Preparation of microspheres

Microspheres were prepared by an emulsification-solvent evaporation method. A certain amount of PLGA was dissolved in dichloromethane, added to the appropriate amount of MLX, ultrasonic 3 min, obtain drug-containing suspension; then added drop by drop to the water phase 1% PVA, and stirred at 1200 rpm for 5 min. Add 0.1% PVA, solidify at 500 rpm/min for 5 h, make the solvent fully volatilize, and screen, the microspheres were collected and washed with distilled water. The resulting microspheres were finally freeze-dried and stored at 4 °C.

### 2.3. Characterization of microspheres

#### 2.3.1. Morphology and particle size

The surface morphology of MLX-MS was observed by SEM (EM-30PLUS, Coxem, Germany). Installation of freeze-dried microspheres on metal columns with sticky carbon strips. The average particle size and size distribution of microspheres were measured by Malvern 3000 laser particle size analyzer (Malvern Instruments Ltd., Malvern, UK), and the dispersion index (Span) was calculated to evaluate the size distribution.

#### 2.3.2. Drug loading and entrapment efficiency

The drug loading (DL) and encapsulation efficiency (EE) of MLX-MS were determined by HPLC. Briefly, accurately weighed 20 mg of microspheres were dissolved in 10 mL of methylene chloride. The sample was diluted 10-fold with mobile phase (methanol: 0.1% formic acid water = 70:30), and 6000 rpm/min centrifugal 5 min, collect supernatant to determine the concentration of MLX with waters e2695 chromatographic system (Waters Corporation, Massachusetts, USA). Agilent TC-C_18_ column was used with a column temperature of 35 °C, the flow rate of 1 mL/min. The detection wavelength was set at 362 nm, the injection volume is 10 μL.

$$\mathrm{DL}（\%）=\frac{\text{amount of drug in microspheres}}{\text{amount of microspheres}}\times 100$$

$$\mathrm{EE}（\%）=\frac{\text{amount of drug in microspheres}}{\text{theoretical mass of drug in microshperes}}\times 100$$

#### 2.3.3. In vitro release study

The *in vitro* release was determined by dialysis. 5 mg MLX-MS were transferred to a dialysis bag (8–14 kDa) and placed in a 50 mL centrifuge tube, adding release medium (0.1% Tween 80 pH 7.4 PBS) 20 mL. The microsphere suspension was shaken horizontally at 60 rpm in a shaker maintained 37 ± 0.5 °C, remove 500ul release medium at different times and immediately add fresh, isothermal, and equal volume release medium. The drug concentration in the release medium was determined by HPLC, and the cumulative drug release (Q) was calculated.

To investigate the mechanism of drug release, the *in vitro* release data was analyzed by fitting zero-order, first-order, Higuchi, and Ritger-Peppas equation models (Brown et al., 2006). Polymer degradation during microsphere release was also monitored by using SEM observation.

#### 2.3.4. Fourier transform infrared (FTIR) and X-ray powder diffraction (XRPD) assays

FTIR and XRPD assays were performed for MLX, PLGA, MLX-MS and the mixture of MLX and PLGA. Fourier transform infrared spectrometer (NICOLET iS10, Thermo Fisher Scientific, USA) was used and the samples were prepared by tableting with KBr at a weight ratio of 1: 100, and the scanning range was 400–4000 cm^−1^. XRPD (D-MAX 2500 X, Rigaku Corporation, Japan) was performed at 10°–80° (2θ), the relative strength is read from the bar graph and corrected to fix the slit value.

### 2.4. Animal model and experimental grouping

Male Wistar rats were injected with sodium iodate (MIA) into the left joint to establish the osteoarthritis model (Hanafy & EI-Ganainy,, 2020). The MIA was dissolved with normal saline and the injection volume was 100 μL (30 mg/mL). After injection, the knee joints of rats were massaged to ensure uniformity.

The model rats were randomly divided into two groups (*n* = 4), namely MLX oral administration group and the MLX-MS intra-articular administration group. Meloxicam 0.5% sodium hydroxymethyl cellulose suspension was prepared and administered by gavage at a dose of 1.35 mg/kg for seven consecutive days. For the oral group, blood was collected from the inner canthus venous plexus at 0.5, 1, 2, 4, 8, 12, 24 h after the first dose and 0.5, 1, 2, 4, 8, 12 h after the last administration. The microspheres group was administered by intra-articular injection with a new needle for each injection at a dose of 9.45 mg/kg once (Conaghan et al., 2018;  Li et al., 2021). Blood was collected at 0.5, 1, 2, 4, 8, 12, 24, 48, 96, 120, and 144 h after administration. The blood samples were centrifuged immediately at 12,000 rpm at 4 °C for 10 min and the plasma samples were then obtained and stored at −80 °C. A portion of the samples were naturally solidified at room temperature for 20 min and centrifuged at 3000 rpm at 4 °C for 10 min. The supernatant was taken and IL-6 and TNF-α inflammatory factors were detected according to the instructions.

### 2.5. Drug quantification of biological samples

UPLC-MS/MS assay was performed to determine drug content in biosamples such as plasma and tissues by using an AB Sciex Triple QuadTM 4500 system (AB SCIEX, USA) connected with Shimadzu LC-30AD via electrospray ionization (ESI) interface. The mobile phase was methanol (A) and 0.1 formic acid water (B) with the following gradient elution: 90%–55% (0–0.5 min); 55%–35% (0.5–3.5 min); 35%–10% (3.5–6.5 min), column temperature 40 °C, flow rate 0.4 mL/min, injection volume 5 μL. Mass spectrometry using electrospray ion source ESI, detection mode is positive ion mode, scanning mode is multiple reaction monitoring (MRM), meloxicam m/z:352.3 →115.2, piroxicam m/z: 332.1 → 94.8.

### 2.6. In vitro and in vivo correlation (IVIVC) analysis

According to previous research (D'Souza et al., 2014;  Laavola et al., 2018), the AUC_t_ was calculated by the trapezoidal area method (Formula 1), and then the *in vivo* absorption rate was obtained by the fractional AUC method (Formula 2).

(1) $${\text{AUC}}_{t_{1}-t_{2}}=[\frac{C_{1}+C_{2}}{2}]\times (t_{2}-t_{1})$$

(2) $$F_{a}(t)=\frac{{\text{AUC}}_{t}}{{\text{AUC}}_{\infty }}$$

Fa(t) is the percentage of drug absorbed within time t, AUCt is the area under the plasma concentration-time curve at time t, and AUC_∞_ is the total area under the plasma concentration-time curve. Linear regression analysis was applied to fit the IVIVC plot and R^2^ was calculated to evaluate the IVIVC.

### 2.7. Biochemical and histological analyses

ELISA assays were performed using a microplate reader (Tecan Spark, Tecan, Switzerland) for the known markers of inflammation in osteoarthritis, such as TNF-α and IL-6 in serum. For histological examination, pathological anatomy was performed on rats. Anatomy of both sides of the joint, trim the surrounding muscles. Samples were fixed in 10% formalin for 24 h, rinsed, and joints were decalcified with 5% formic acid for 72 h. Then the samples were dehydrated, embedded in paraffin, sliced and stained with HE, and observed under light microscope for histological evaluation. Furthermore, referring to the Osteoarthritis Research Society International OARSI scoring criteria (Table 1), we evaluated the histology of osteoarthritis in rats (Gerwin et al., 2010).

**Table 1.** OARSI histopathological evaluation criteria.

Histological feature	Score
Cartilage degeneration	
Normal	0
Minimal degeneration	1
Mild degeneration	2
Moderate degeneration	3
Marked degeneration	4
Severe degeneration	5
Calcified cartilage and subchondral bone damage	
Normal	0
No fragmentation of tidemark; no marrow changes	1
Mesenchymal change in marrow	2
Mesenchymal change in marrow; areas of marrow chondrogenesis may be evident	3
Marked to severe fragmentation of calcified cartilage; marrow mesenchymal change involves up to 3/4 of area	4
Synovial membrane inflammation	
Normal	0
Slight proliferation of subsynovial tissue.	1
Proliferation of subsynovial tissue	2
Proliferation of subsynovial tissue; infiltration of few inflammatory cells	3
Proliferation of subsynovial tissue; infiltration of large number of inflammatory cells.	4

### 2.8. Statistics

One-way analysis of variance (ANOVA) and two-tailed Student’s t-test was performed for significance tests. A p-value less than 0.05 was considered statistically significant.

## 3. Results and discussion

### 3.1. Optimization of microsphere formulation

#### 3.1.1. Primary screening of PLGA

A single-factor test was used to screen the type of PLGA. According to the preparation method above, MLX-MS were prepared with different kinds of PLGA. The particle size, drug loading (DL), encapsulation efficiency (EE), and span of microspheres are shown in Table 2. Cluster analysis is carried out according to the particle size, drug loading, and encapsulation efficiency of microspheres by the hierarchical cluster method, the nine PLGAs were clustered into three categories, PLGA 50502E and 95052A were classified into one category, 50502A and 75254A were classified into one category, and the remaining five categories were classified into one category.

**Table 2.** Characterization of microspheres prepared by different kinds of PLGA.

PLGA	Particle size(μm)	DL(%)	EE(%)	Span
5050 1A	99.2 ± 5.05	23.07 ± 0.07	63.27 ± 0.19	0.519
5050 2A	104.9 ± 6.25	13.59 ± 0.08	55.77 ± 0.33	0.542
5050 4A	112.0 ± 6.32	17.22 ± 0.43	59.87 ± 1.54	0.471
5050 2E	102.0 ± 1.67	23.84 ± 0.07	99.53 ± 0.33	0.912
7525 1A	109.0 ± 0.07	22.57 ± 0.03	68.60 ± 0.08	0.632
7525 2A	106.0 ± 3.82	19.86 ± 0.01	68.10 ± 0.01	0.408
7525 4A	138.0 ± 6.94	14.47 ± 0.29	73.50 ± 1.50	0.510
8515 2A	108.0 ± 6.10	19.54 ± 0.05	60.10 ± 0.16	0.473
9505 2A	111.0 ± 2.41	22.99 ± 0.09	102.10 ± 0.41	0.448

In summary, considering the encapsulation efficiency, drug loading, 50502A, 50502E, and 75252A were finally selected from the three categories for further screening.

#### 3.1.2. Orthogonal test

An orthogonal experiment was used to optimize the formulation of MLX-MS. Referring to the results of the single factor experiment, PLGA type, PLGA/MLX, PLGA concentration, and O/W were selected to optimize the formulation by L_9_(3)^4^ orthogonal design.

**Figure 1.**
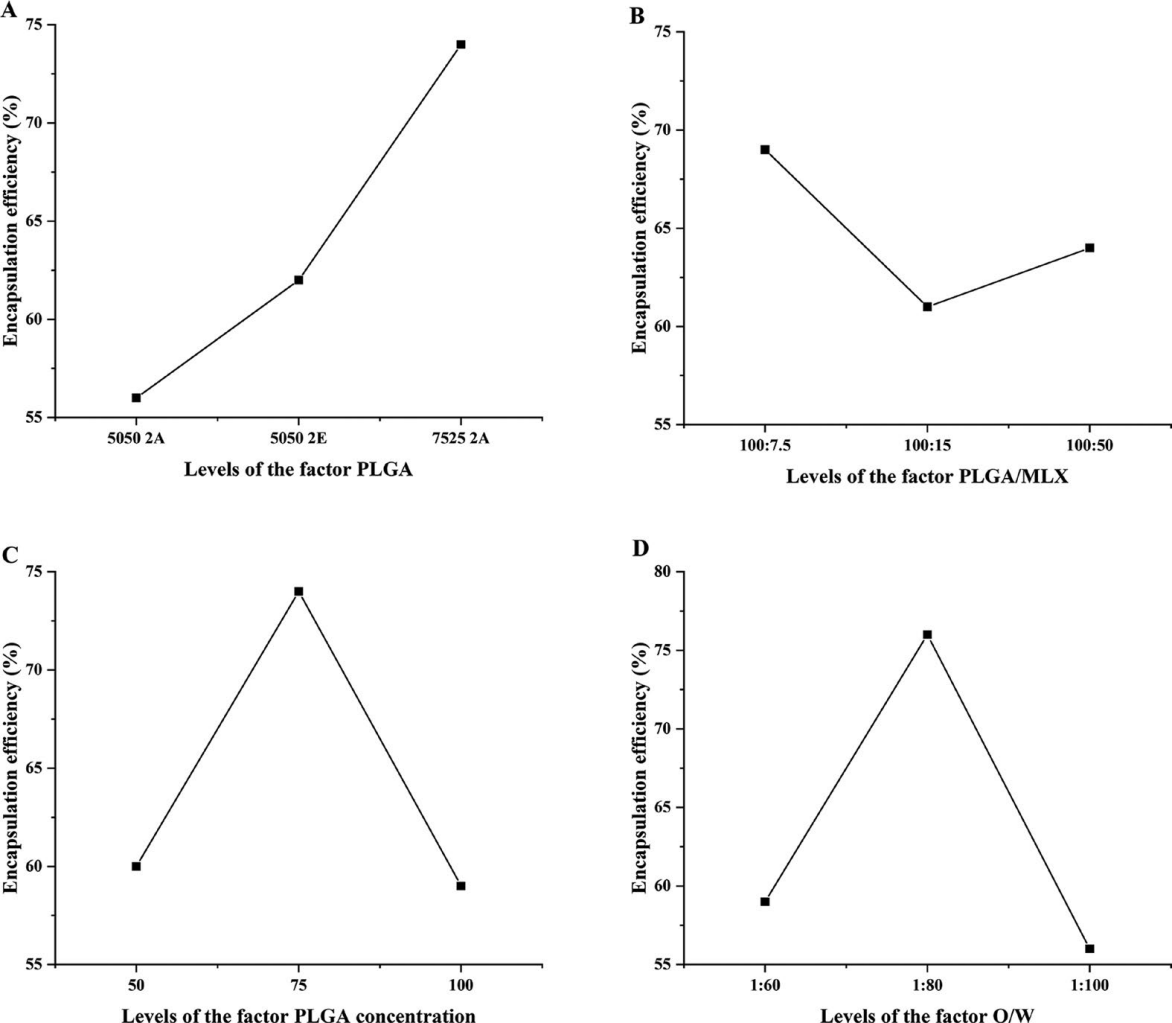
The impact trend of PLGA (A), PLGA/MLX (B), PLGA concentration (C), and O/W(D) on encapsulation efficiency of microspheres.

The factor level table of the orthogonal test is shown in Table 3, and the three-factor three-level orthogonal table is shown in Table 4. The optimal prescription was screened with the level of encapsulation efficiency as the index. The results showed that the type of PLGA, PLGA/MLX, PLGA concentration, and O/W had significant effects on encapsulation efficiency (*p* < .01). According to the effect of various factors on the encapsulation efficiency (Figure 1), the optimal prescription is A_3_, B_1_, C_2_, and D_2_. To obtain higher drug loading and combined with the results of intuitive analysis, A_3_, B_3_, C_2_, and D_2_ was the optimal prescription, namely PLGA type was 7525 2A, PLGA/MX was 2:1, PLGA concentration was 100 mg/mL, O/W was 1:80.

**Table 3.** Orthogonal design and experimental results.

Levels	Factor			
	A	B	C	D
1	5050 2A	100:7.5	50	1:60
2	5050 2E	100:15	75	1:80
3	7525 2A	100:50	100	1:100

Factor A: PLGA, factor B: PLGA/MLX(m/m), factor C: Concentration of PLGA (mg/mL), factor D: O/W (v/v).

**Table 4.** Orthogonal design and experimental results (*n* = 3).

No.	A	B	C	D	Particle size(μm)	EE(%)	DL(%)	Span
1	1	1	1	1	111 ± 0.01	52.71 ± 0.17	5.4 ± 0.02	0.430
2	1	2	2	2	127 ± 1.90	74.63 ± 0.67	12.8 ± 0.12	0.467
3	1	3	3	3	156 ± 4.80	42.63 ± 0.02	33.1 ± 0.02	0.548
4	2	1	2	3	143 ± 5.90	70.18 ± 0.49	6.6 ± 0.05	0.493
5	2	2	3	1	142 ± 0.44	46.11 ± 0.24	8.5 ± 0.04	0.479
6	2	3	1	2	109 ± 1.50	70.51 ± 0.25	37.8 ± 0.13	0.446
7	3	1	3	2	127 ± 8.50	86.62 ± 0.77	7.8 ± 0.07	0.489
8	3	2	1	3	111 ± 1.80	56.08 ± 0.11	12.3 ± 0.12	0.467
9	3	3	2	1	125 ± 7.60	79.19 ± 0.27	37.6 ± 0.38	0.492

**Figure 2.**
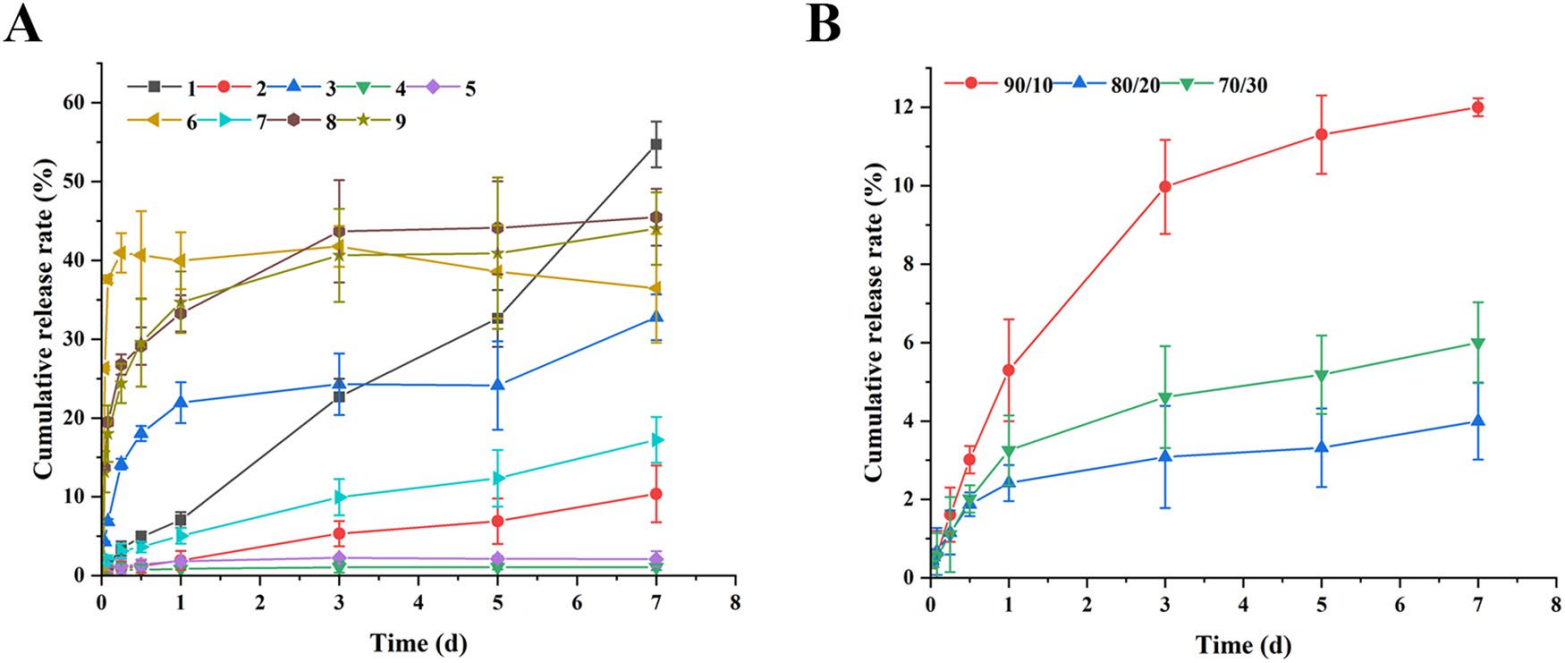
The *in vitro* release profiles of (A) microspheres prepared by different polymers, and (B) PLGA blends microspheres.

The *in vitro* release characteristics of the nine prescriptions were also investigated, and the results were shown in Figure 2(A). Different types of PLGA have a great influence on the drug release characteristics of microspheres. For the microspheres prepared by PLGA 5050 2E, MLX was tightly wrapped inside the microspheres and could not be released, the reason was that MLX was a hydrophobic drug and had a strong binding force with PLGA 5050 2E ester-terminated polymer, so it was easier to combine with MLX; Moreover, the slower degradation rate of ester-terminated PLGA also limited the release of drugs in the microspheres (Panyam et al., 2004; Mao et al., 2007; Félix Lanao et al., 2011). However, about 40% of the drug was released in prescription 6, which was speculated the dissolution of some unencapsulated drug on the surface of the microspheres due to the high loading

The PLGA 7525 2A MLX-MS release slowly, because the proportion of lactide (LA) increases, which reduces the degradation rate of PLGA itself, and the interaction between the drug and PLGA increases with increasing dose, resulting in a slower release rate (Schliecker et al., 2003; Siegel et al., 2006). From the results of the experiment, we can find that formulations 6, 8, and 9 have obvious burst release at the initial stage of release. It’s speculated that the drug adsorbed on the surface of the microspheres is dissolved in the release medium to form a burst release, or the drug is not uniformly dispersed, drug outside the microspheres triggers burst release (Leach et al., 2005; Lee et al., 2010).

#### 3.1.3. Microspheres based on PLGA blends

From the release curves of different formulations, the microspheres prepared by PLGA 50502A have sustained-release properties *in vitro*, but the encapsulation efficiency is low. To obtain microspheres with higher drug loading and improved drug release characteristics, we mixed PLGA 5050 2A and 7525 2A in a certain proportion (90/10, 80/20, 70/30), and other parameters following the above optimization results.

The *in vitro* release results were shown in Figure 2(B), MLX-MS release behavior significantly improved after mixing two different PLGA. When the proportion of PLGA 7525 2A increased, the microspheres were not released after three days, and with the increase of 5050 2A, the speed of drug release is gradually accelerated, this is because PLGA blends affected the internal structure of the microspheres (Mao et al., 2007). In summary, the optimal process is PLGA type 5050 2A (90%)/7525 2A (10%), PLGA/MX 2:1, PLGA concentration 100 mg/mL, and O/W 1:80.

### 3.2. Characterization

#### 3.2.1. Morphology and particle size

To further verify the reliability of the optimal prescription, six batches of MLX-MS were prepared with the optimal prescription. The drug loading was 36.77 ± 3.5%, the encapsulation efficiency was 76.81 ± 7.13%, the particle size was 102 ± 3.90 μm, and the particle distribution was uniform.

**Figure 3.**
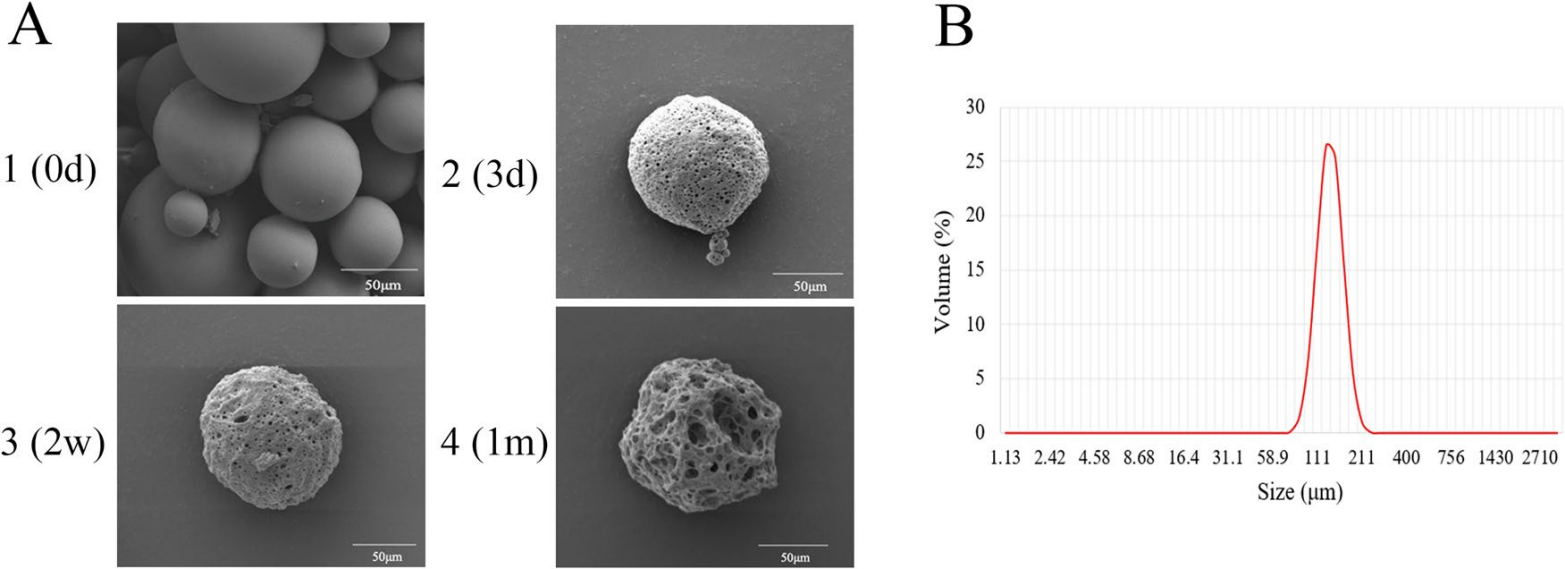
SEM observation (A) and particle size distribution (B) of MLX-MS.

Scanning electron microscopy (SEM) was used to investigate the morphology of MLX-MS. The results were shown in Figure 3(A-1). The microspheres were spherical in shape, smooth and non-porous on the surface, and there is no adhesion between the microspheres, indicating that the microspheres have a good distribution. The results of particle size determination of multiple batches of microspheres by dynamic light (DLS) scattering showed (Figure 3B) that the average particle size of the microspheres was between 100 and 110 μm, and the span was 0.5–0.6, indicating that the distribution of the microspheres was uniform.

#### 3.2.2. Spectral analysis

**Figure 4.**
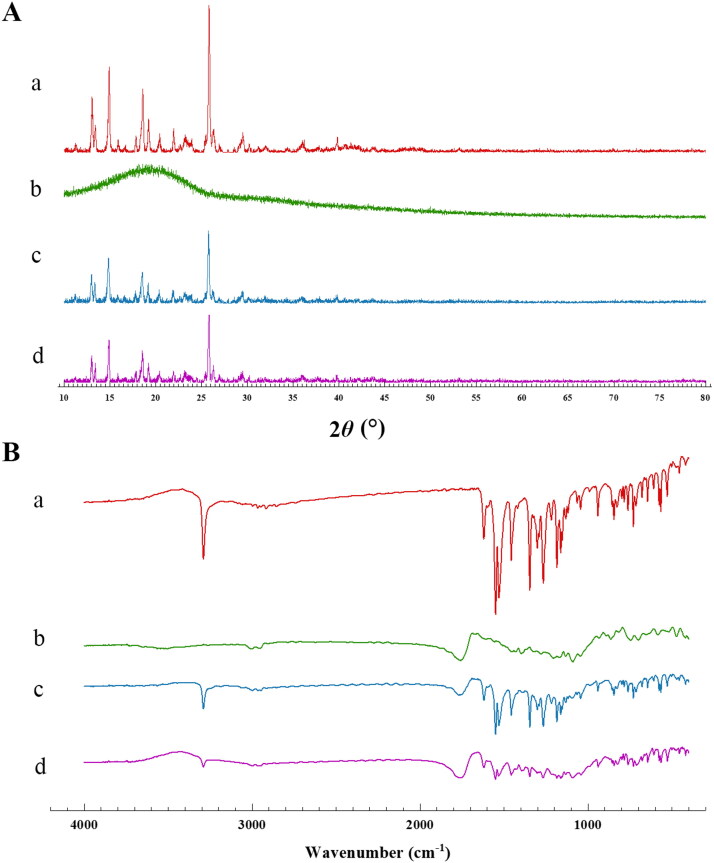
XRPD (A) and IR (B) spectra (a: PLGA, b: MLX, c: PLGA/MLX, d: MLX-MS).

Meloxicam is a polymorph, so during the preparation of microspheres, including dissolution and desolvation processes, the crystallinity of MLX will be affected. We used the MLX/PLGA physical mixture with the same material composition as a reference and analyzed the XRPD and IR spectra under the same conditions. The characteristic peaks in the spectrum were used as indicators to investigate whether the dissolution and solvent removal process during the preparation of the microspheres affected the crystal form of the raw material. The results were shown in Figure 4(A). The peak of MLX is about 25.8°, and the broad peak of PLGA is about 19.6°. It can be seen that the characteristic peaks of MLX exist in the microspheres and the mixture of PLGA/MLX, although the response is reduced, the peaks were not broadened or shifted. The FTIR results were shown in Figure 4(B). MLX shares characteristic absorption peaks at 3290.56 cm^−1^, and PLGA shares characteristic absorption peaks at 3002.41 cm^−1^ and 2954.78 cm^−1^, these characteristic peaks exist in the physical mixture of MLX-MS and PLGA. The presence of these peaks indicates that the drug did not react with the polymer and that the drug was encapsulated in the microspheres (Erdemli et al., 2014). Therefore, it was considered that MLX was encapsulated in PLGA and the structure did not change.

#### 3.2.3. In vitro drug release

**Figure 5.**
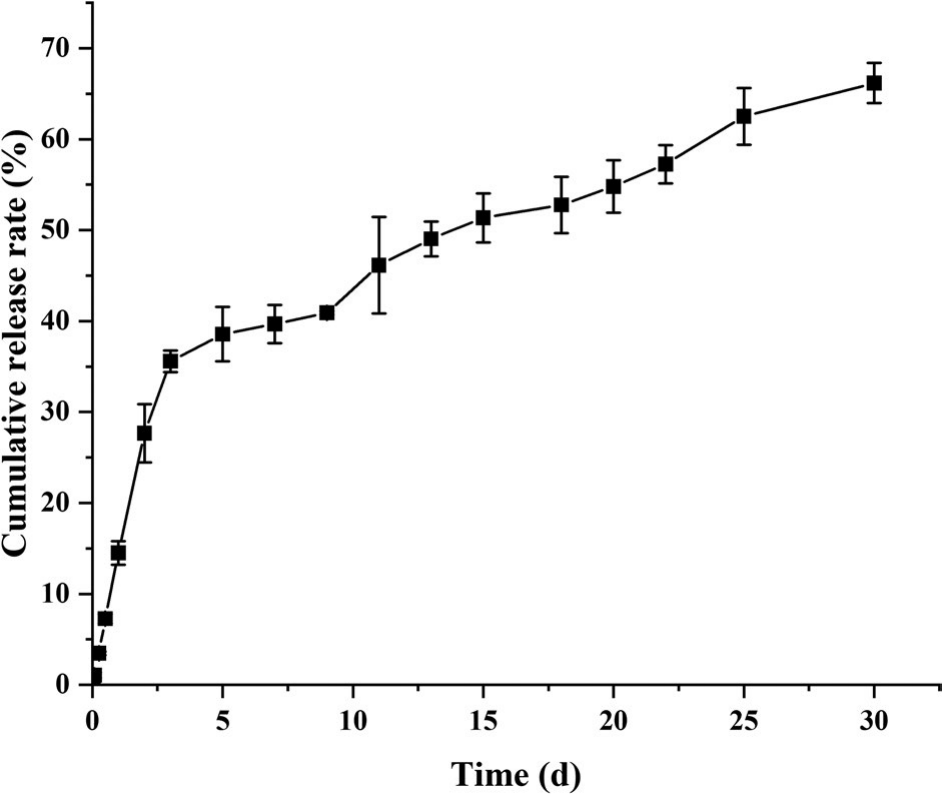
The *in vitro* release profile of MLX-MS obtained under optimal conditions (n = 3).

The *in vitro* cumulative release curves of MLX-MS were shown in Figure 5. The results showed that the release of MLX-MS was biphasic, MLX rapid release within 72 h, then entered the stage of slow drug release. The rapid release is due to the increased drug concentration near the surface caused by solvent convection during the preparation of the microspheres, so the drug is rapidly released in the form of diffusion (Allison, 2008). The second release stage is mainly controlled by the degradation of PLGA. Drug release is due to the gradual hydrolysis of PLGA to oligomers, which leads to the formation of pores on the surface and inside of microspheres, providing conditions for drug release through diffusion and erosion within microspheres (Makadia & Siegel, 2011).

The mechanism of MLX release from the microspheres was investigated by fitting the data obtained from *in vitro* release (Table 5). The *in vitro* release of MLX-MS follows the Ritger-Peppas model, (R^2^ = 0.9969). The drug release characteristic index ‘n’ in the release curve of the Ritger-Peppas equation is analyzed, when *n* ≤ 0.45, the drug release mechanism is Fick diffusion, and when 0.45 < *n* < 0.89, the drug release mechanism is the combined effect of drug diffusion and matrix erosion. When *n* ≥ 0.89, the drug release mechanism is skeletal erosion. Therefore, the drug release mechanism of MLX-MS would be the coexistence of drug diffusion and skeletal erosion (*n* = 0.46) (Siepmann et al., 2005). The degradation results of the microspheres were shown in Figure 3(A2–4). The microspheres were continuously degraded in the release system. In the initial stage of release (3 days), there were tiny holes on the surface. After 2 weeks, the size of the holes increased and the microspheres gradually dissolved. Around 1 month, the microspheres rupture. Thus, with the continuous degradation of PLGA, the structure of microspheres changed greatly. In this process, the microspheres continue to dissolve, the pores increase, and the drug is gradually released from the microspheres.

**Table 5.** The kinetic models simulated for the release behavior of microspheres.

Model	Equation	R^2^
Zero-order model	${\text{ Q}}_{t}=0.0887t+13.8271$	0.8020
First-order model	${\text{ Q}}_{t}=56.2160-e^{-0.0094t}$	0.9522
Higuchi model	${\text{ Q}}_{t}=2.5312t^{1/2}+2.5168$	0.9495
Ritger-Peppas model	${\text{ Q}}_{t}={3.2202t}^{0.4683}$	0.9969

### 3.3. Therapeutic effects

**Figure 6.**
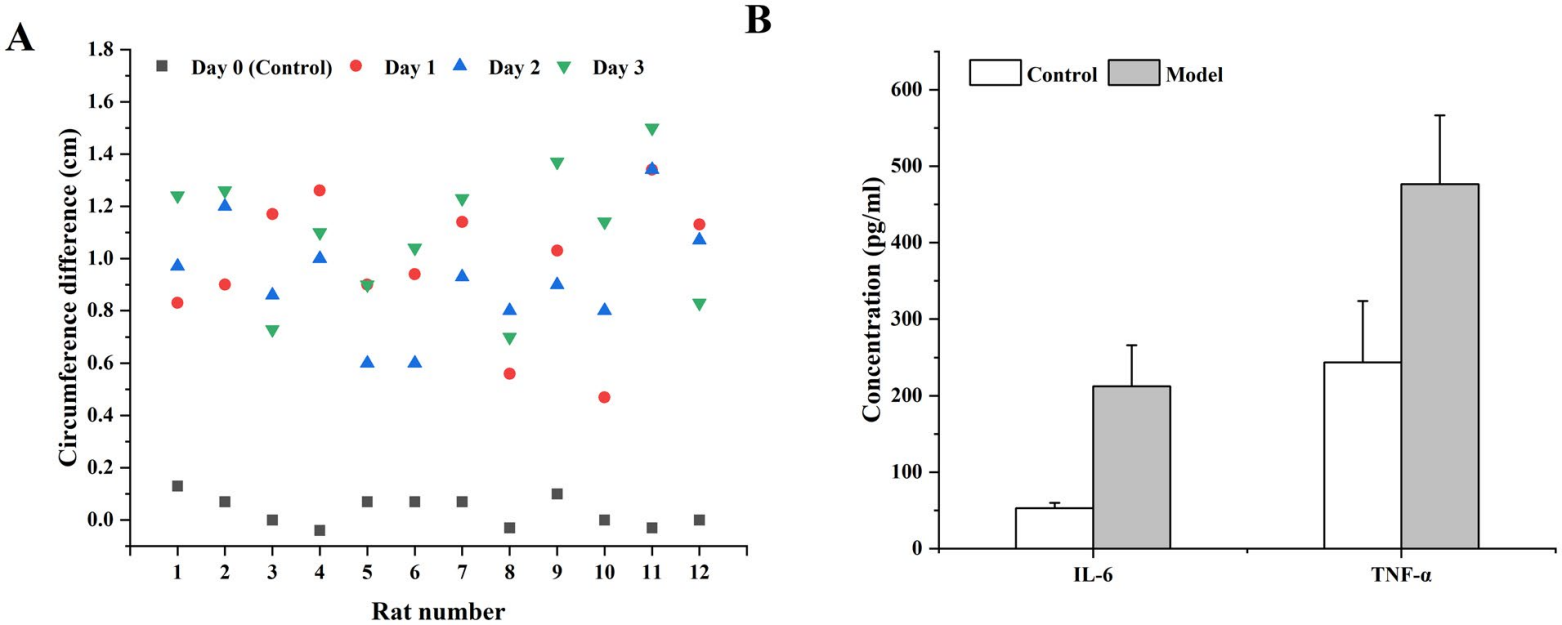
Changes of periarticular length difference (A) and inflammatory factors (B) in rats before and after modeling (**p < 0.01, compared with control).

The results of changes in the circumference of the knee joint of the rats were shown in Figure 6(A). The right leg of the rat was used as a control to monitor the change of the difference in the circumference of the left after the injection of MIA. The results showed that the swelling degree of the left joint of the rats increased significantly with time, and the difference in the circumference of the joints on both sides showed a continuous and significant change with time, indicating that the inflammation model was successful. To further study the efficacy of MLX-MS *in vivo*, this experiment also investigated the changes in inflammatory factors in rats.

It has been reported that a variety of inflammatory factors were involved in the occurrence of OA, such as IL-1β, IL-6, TNF-α, etc., which were closely related to the degeneration of articular cartilage and the production of OA (Chu et al., 2006; Kapoor et al., 2011). We evaluated the changes of inflammatory cytokines IL-6 and TNF-α in rats after oral administration and microsphere injection. The results were shown in Figure 6(B). Compared with the blank control group, the levels of inflammatory factors IL-6 and TNF-α in the rats after MIA injection were significantly increased (*p* < .01), indicating that the experimental arthritis model was successful.

**Figure 7.**
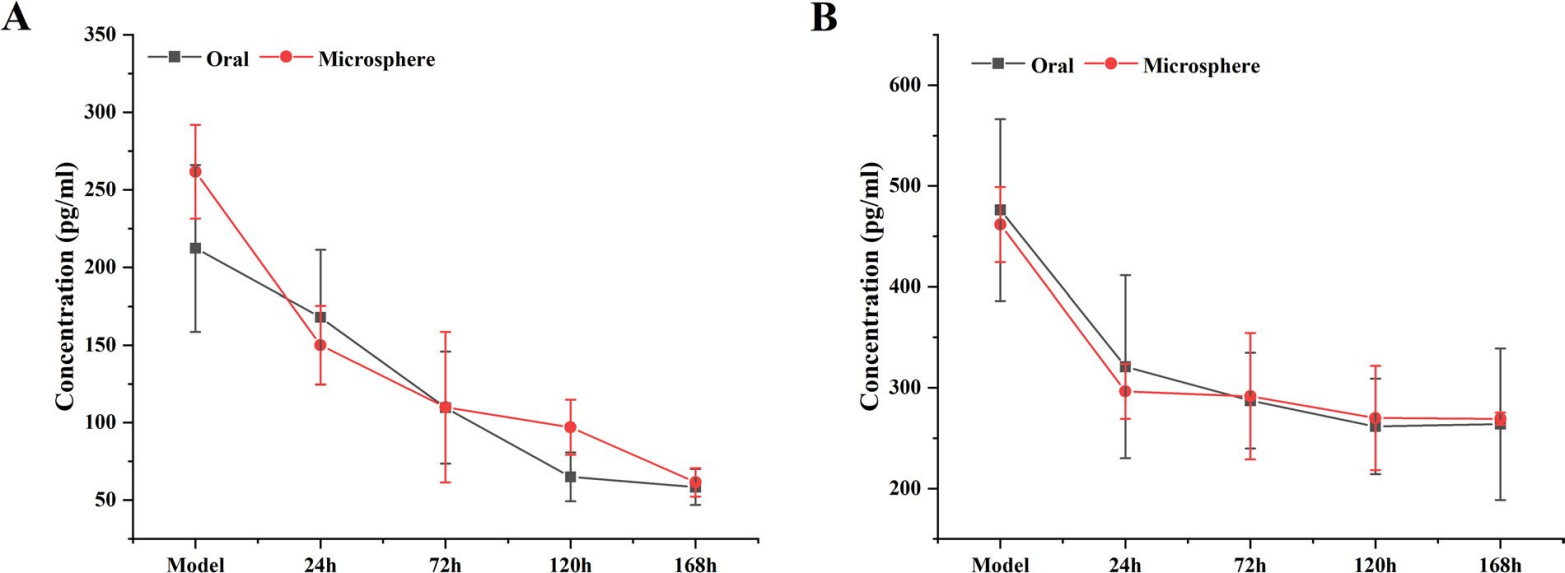
Changes of inflammatory factor IL-6 (A), and TNF-α (B) in rats (n = 4).

Within seven days of oral administration and microsphere injection, the levels of IL-6 and TNF-α in rats gradually decreased (Figure 7). For the MLX-MS intraarticular injection, the drug is released in the joint cavity and quickly reaches the site of inflammation to exert its efficacy. Moreover, due to the slow release of the drug, the level of inflammatory factors in the rat body decreases at a lower rate after 72 hours than in the oral administration group. Feature-related pharmacodynamic relationships were more pronounced on IL-6, which could be further analyzed by PK-PD.

### 3.4. Pharmacokinetics in plasma

#### 3.4.1. Drug concentration-time curve and pharmacokinetic parameters

**Figure 8.**
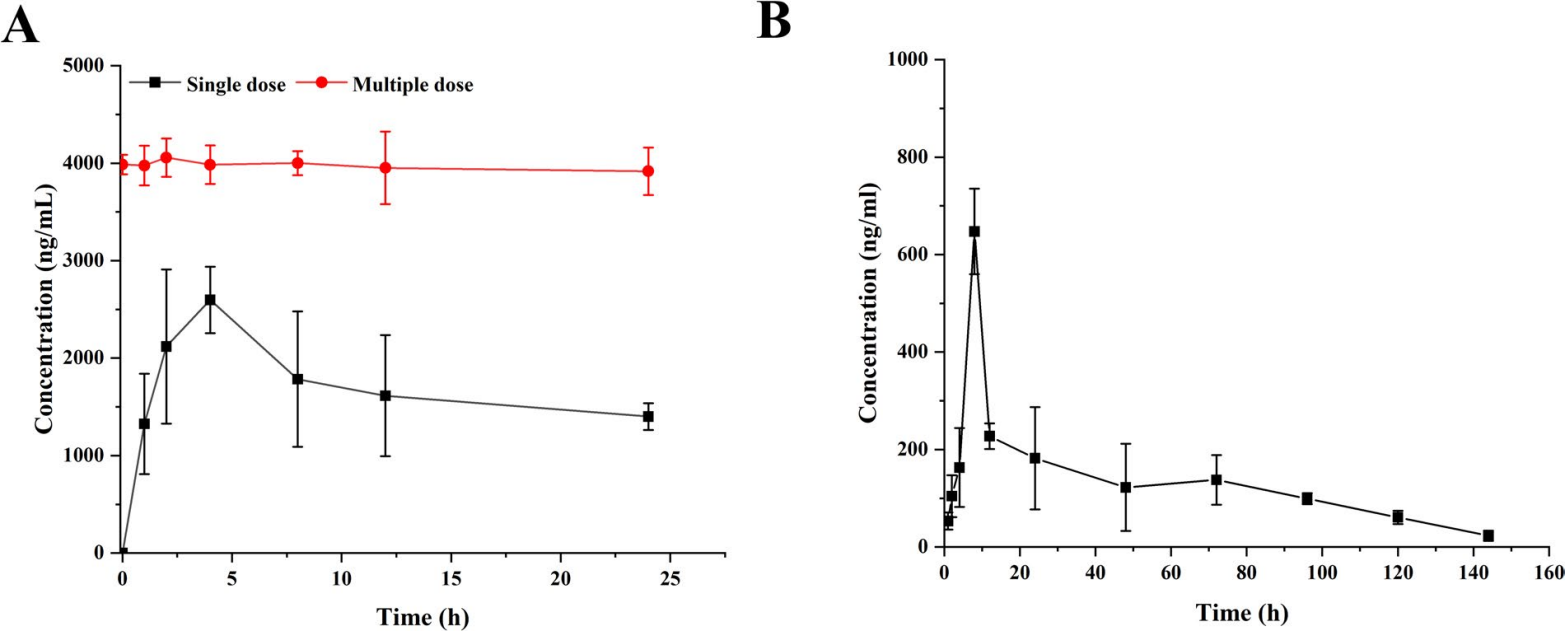
Mean plasma concentration-time profile of MLX in rats (A) oral administration, and (B) injection of microsphere (n = 4).

From the results in Figure 8, it can be seen that the plasma drug concentration during 0.5–12 h, plasma drug concentration in the oral group at each time point was significantly higher than that in the microsphere group, and soon reached the maximum value; after continuous oral administration, MLX reached a steady-state concentration *in vivo*. After the MLX-MS were injected with the drug solution into the joint cavity, the drug leaked through the joint cavity and was absorbed into the blood, so compared with the solution group, the blood drug concentration was lower.

Its main pharmacokinetic parameters were shown in Table 6. After oral administration of MLX, the plasma concentration reached its peak at 4 hours, and the C_max_ was 2.60 ± 0.39 μg/mL. The time for the peak plasma concentration of the microspheres was 8 hours, and the C_max_ was significantly decreased to 0.78 ± 0.76 μg/mL. Compared with oral administration, the T_1/2_ microspheres were prolonged, which was about 1.1 times that of oral administration; AUC_0-∞_ after oral administration was 5.14 times higher than microspheres administration. It can be seen that the blood drug concentration of microsphere administration is much lower than that of oral administration, thereby reducing the systemic side effects caused by high blood drug concentration. The administration of microsphere injection can effectively achieve the effect of sustained drug release.

**Table 6.** Pharmacokinetic parameters.

Parameter	Oral		Microsphere
	Single dose	Multiple dose	
C_max_ (μg/mL)	2.77 ± 0.21	2.60 ± 0.39	0.78 ± 0.76*
T_max_ (h)	4 ± 0.00	4.00 ± 0.00	8.00 ± 0.00*
AUC_0–∞_ (μg/L*h)	117.28 ± 4.10	95.73 ± 54.56	22.81 ± 9.29*
MRT_0–∞_ (h)	52.40 ± 22.15	48.12 ± 20.00	56.11 ± 11.41
T_1/2_ (h)	32.64 ± 18.72	30.80 ± 15.50	33.28 ± 4.29

AUC, area under the curve; MRT, mean residence time.

*p < .05 compared with the oral administration group.

#### 3.4.2. IVIVC

The *in vitro* and *in vivo* correlation is always a crucial issue for the dosage forms in which the drug needs to be absorbed into the circulation, since it is important for both formulation development and quality control (Sun et al., 2008). *In vitro* and *in vivo* correlations can be divided into three categories, among which the point-to-point relationship is classified as an A-level correlation by the US Food and Drug Administration (Ma et al., 2020). In this study, a point-to-point relationship between *in vitro* drug release and *in vivo* drug absorption was observed for MLX-MS. There was a good linear relationship between the fraction of MLX absorbed *in vivo* and the percent released *in vitro*: y = 3.6043x − 16.8842 (R^2 ^=0.9945). Therefore, we conclude that MLX-MS exhibited similar release profiles *in vitro* and *in vivo*, and it seems reasonable to predict *in vivo* drug absorption from *in vitro* release studies.

#### 3.4.3. PK-PD correlation

Comparison of MLX pharmacokinetics at corresponding time points with IL-6 levels. First, from the time-concentration-effect curves we can see that when the blood concentration of MLX reaches C_max_, the inflammatory factors in the rat body decrease the fastest. Intra-articular injection allows MLX to quickly reach the site of inflammation to play a role, so although the plasma concentration decreases, the level of inflammatory factors remains decreased. Further bivariate correlation analysis was performed. The results show that a positive correlation between PD-PK and the correlation coefficient is 0.981 (*p* < .05).

#### 3.4.4. Tissue distribution and histopathology

**Figure 9.**
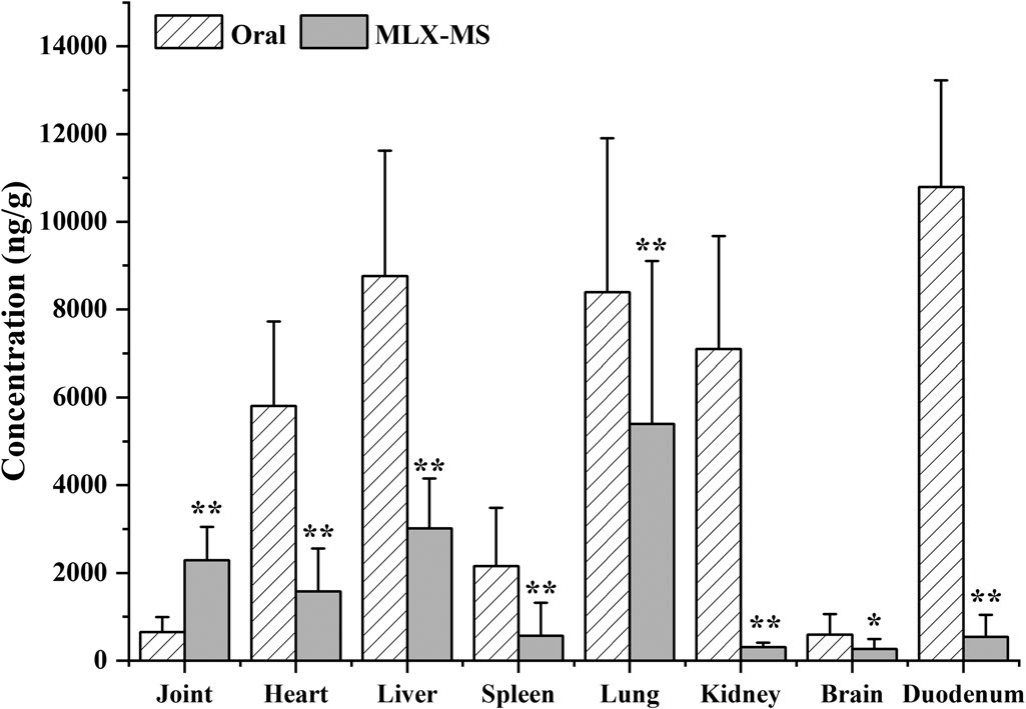
Tissue distribution of MLX in rats (n = 4, **p < 0.01, compared with oral group).

As shown in Figure 9, MLX is widely distributed in the heart, liver, spleen, lung, and kidney of rats after oral administration and joint injection of MLX-MS. In the heart, liver, spleen, lung, kidney, brain, the drug distribution of the MLX-MS group was lower than that of the MLX oral administration group (*p* < .05), while in joint tissue, the distribution of the MLX-MS group was significantly higher than MLX oral administration group (*p* < .01). It can be seen that after intra-articular administration, the drug can be quickly concentrated in the joint and its surrounding tissues, and play efficacy faster. At the same time, the distribution of drug in duodenum decreased significantly (*p* < .01) after microsphere injection, which can effectively reduce the gastrointestinal side effects of MLX.

**Figure 10.**
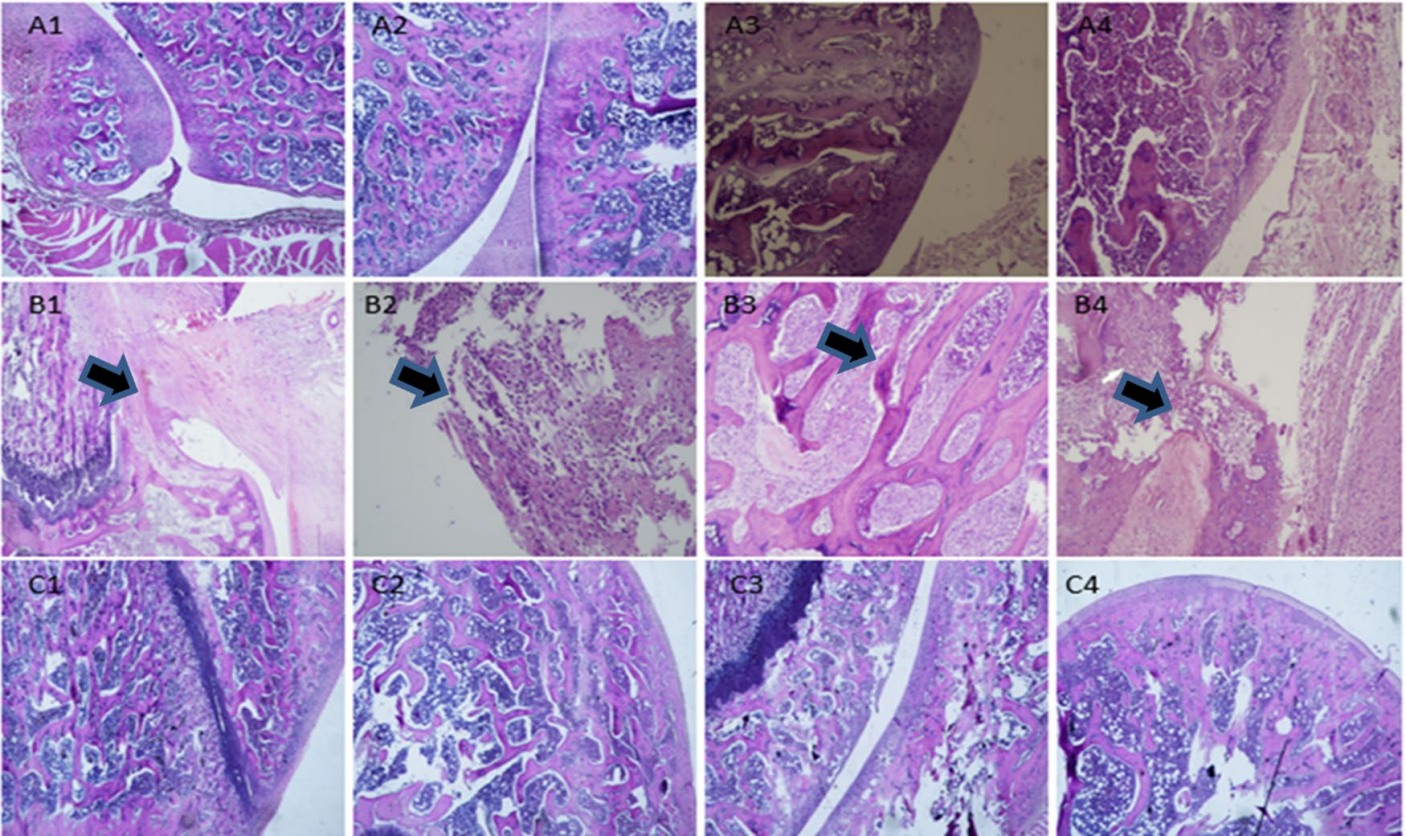
Pathological sections (×100) in blank control group (A1-A4), osteoarthritis model group (B1-B4), and MLX-MS group (C1-C4).

The histopathological examination results of rats were shown in Figure 10. According to the histopathological scoring results shown in Table 7, the score of model group was higher than that of blank control group. The articular cavity of normal rats (A1–A4) maintained a steady state, the surface of the articular cartilage was smooth and flat, and the chondrocytes were evenly distributed and arranged neatly. Occasionally, chondrocyte hypertrophy was observed, and no obvious cell clustering was observed. In the Model group (B1–B4), there were different degrees of defects in the left articular cavity, soft tissue edema tissue in the articular cavity, focal necrosis and hemorrhage, infiltration of inflammatory cells, formation of foam cells and multinucleated giant cells, and proliferation of synovial blood vessels. In addition, the articular cartilage of rats became thinner and distributed disorderly. A large number of hypertrophic chondrocytes were seen in the bottom of the cartilage, and some articular cartilage was hardened. The proliferation of bone marrow cells in the bone marrow cavity was active, showing obvious symptoms of arthritis. As shown in Figure 10(C1–C4), Histological score of OA decreased significantly after MLX-MS intervention (*p* < .05), rat bone tissue cell regeneration, partial recovery of bone hyperplasia; there was no obvious tissue necrosis and hemorrhage in the joint cavity. The edema of the surrounding soft tissue disappeared. The fibrous tissue proliferated. There was no obvious inflammatory cell infiltration.

**Table 7.** Rat histopathological score.

Group	Histological feature		
	Cartilage degeneration	Calcified cartilage and subchondral bone damage	Synovial reaction
Blank control	0	0	0
Model	4	4	4
MLX-MS	1*	0*	2

*p < .05 compared with the Model group.

## 4. Conclusion

In this study, a novel MLX-MS for local injection was developed for the treatment of arthritis, and the microspheres with high drug loading and encapsulation efficiency were obtained by using PLGA blends (PLGA 5050 2A and PLGA 75525 2A). The in vitro evaluation results show that MLX-MS has good sustained-release behavior. The in vivo effect evaluation shows that the microspheres can reach the site of inflammation faster after being injected into the joint cavity, and reduce the distribution of the drug in the intestinal tract, effectively reducing the risk of intestinal side effects. Therefore, such new dosage forms were expected to prolong the treatment time, reduce the number of administrations, improve patient compliance, and have good application prospects.
